# Blood in the Urine—Some Cases

**Published:** 1909-02

**Authors:** Alfred L. Fowler

**Affiliations:** Atlanta, Ga.; Professor of Genito-Urinary Surgery and Venereal Diseases in the Atlanta College of Physicians and Surgeons; Genito-Urinary Surgeon to the Grady Hospital; Physician and Surgeon to the United States Penitentiary Hospital; Surgeon to St. Joseph’s Infirmary, Etc.


### BLOOD IN THE URINE—SOME CASES.*

BY ALFRED L. FOWLER, M. D., ATLANTA, GA.

PROFESSOR OF GENITO-URINARY SURGERY AND VENEREAL DISEASES IN THE ATLANTA COLLEGE OF PHYSICIANS AND SURGEONS; GENITO-URINARY SURGEON TO THE GRADY HOSPITAL; PHYSICIAN AND SURGEON TO THE UNITED STATES PENITENTIARY HOSPITAL; SURGEON TO ST. JOSEPH'S INFIRMARY, ETC.

The physician who desires to gain a keener insight into the urinary hemorrhage can only acquire it by the careful thought and study necessary to comprehend its true meaning. The ability to manage it, together with the many perplexing conditions that frequently arise, becomes much easier when a thorough knowledge of all its causes and details are mastered; so that the labor required to control it is fully bestowed. Hematuria is the most important objective symptom of the genito-urinary tract and is common to many of the grave affections involving the genito-urinary organs.

Blood appears in the urine as the result of several distinct and separate affections of the uro-genital tract. As examples:

1.—External trauma (a blow from without) may so damage the kidney, the prostate, the testicles or the penis sufficiently to cause hemorrhage of these organs which subsequently finds its way into the urine. Internal trauma to the uro-genital membrane such as the passage of the renal calculus, the movements of a large vesical calculus, clumsy instrumentation in catheterization or cystoscopy on the part of a neophyte, may also give rise to it.

2.—Local diseases, such as acute Bright's, cancer, sarcoma,

*Read before the Fulton County Society of Medicine, January 7, 1909.

parasitic diseases, such as *Bilharzia haematobia* and particularly gonorrhoea and tuberculosis; drugs, genign villous tumors, hy-
gonorrhoea and tuberculosis; drugs, benign villous tumors, hy-

3.—Constitutional or circulatory diseases dependent on depravity or disorganization of the blood, such as pernicious malaria, scorbutus, purpura hemorrhagica, typhus fever and syphilis.

4.—Vaso-motor disturbances to the sympathetic system—persistent active hyperaemia and, very rarely, passive congestion concomitant to obstructive cardiac lesion.

While the presence of blood in the urine is not, of itself, an essential disease, it being merely a symptom, we should remember that occasionally some of its causes are so thoroughly veiled in obscurity that we find ourselves unable to detect the precise pathologic condition upon which much depends. Particularly is this true if we are not thoroughly familiar with all its probable causes. It is only by careful thought and study of all the available data at our command that we can arrive at a correct diagnosis.

Besides the valuable data obtained by interrogating the patient, which will give us a presumptive diagnosis, it behoves us to verify our suppositions by resorting to the exact methods of examination.

The most orderly way of beginning an examination is to take an accurate anamnesis. We endeavor to learn just what diseases have occurred in the patient's family and of what particular maladies his near relatives died. This inquiry may disclose the fact that tuberculosis, rheumatic and gouty affections, or lithiasis occurred in the patient's family because these diseases play an important role in heredity.

From the patient himself we must endeavor to learn, besides his age, nativity and temperament, whether he has ever had gonorrhoea, syphilis, scarlet fever, hemorrhoids, traumatic injury or other significant diseases.

Having secured all the information obtainable concerning the patient's present illness, its mode of onset, its course and duration, the following detailed information should be sought with the object of at least arriving at a presumptive diagnosis, to be verified later:

When was blood first observed in the urine?

Was the blood clotted or in solution?

Has the blood in the urine ever completely disappeared at any time? If so, how many days at a time?

Does the hemorrhage always take place under the influence of motion? (Note 1).

Does bleeding occur suddenly? (Note 2).

Does it come on without apparent cause and last a long time? (Note 2).

Is the hemorrhage difficult to control by treatment? (Note 2).

Does the influence of rest have any apparent effect on the hemorrhage? (Note 3).

Does hemorrhage appear at the end of urination? (Note 4).

Is the urine smoky and is the blood uniformly diffused through it? (Note 5).

Are there any hemorrhoids present?

Does the first glass of urine expelled contain clots? (Note 6).

If so, is the blood coagulum dark and firm? (Note 6).

Is there little or no blood visible until urination is about completed? (Note 6).

Does bladder instrumentation increase the hemorrhage? (Note 7).

Do solutions of aluminum sulphate, silver nitrate, antipyrine or adrenalin check the hemorrhage? (Note 7).

Does the first glass of urine expelled contain blood? (Note 8).

What is the difference in the relative quantity and quality of blood contained in the first, second, or third glasses?

Is urination more frequent than formerly?

Is the urgency of urination present both day and night?

Is the desire to urinate more frequent in the day than at night?
(Note 9).

Does rest exert any influence upon frequency of urination? (Note 10).

Does activity during the day seem to increase the number of urinations? (Note 10).

Is the quantity of urine passed normal and urination frequent? (Note 11).

Is the quantity of urine passed larger than formerly? (Note 12).

Does the patient sleep all night without having to urinate? (Note 13).

Is the desire to urinate more frequent at night? (Note 14).

Is the urine expelled in an arched stream or does it fall perpendicularly? (Note 14).

Is the urination ever suddenly arrested?

It there ever any pain over the bladder?*

Is there ever any pain in the region of the kidneys? (Note 15).

Is there ever any pain in the urethra?

If in the urethra, is it near the end of the penis or in the deeper portions of the urethra? (Note 10).

Does the pain follow urination? (Note 10).

Does the pain last some time thereafter? (Note 16).

Does pain occur independently of urination? (Note 10).

Does exercise increase the pain?

Does rest lessen the pain?

Is there often felt a dull, aching pain in the perineum and rectum? (Note 14).

Is pain felt only upon urination? (Note 17).

Additional information, other than that gained by interrogating the patient, may be had by a thorough examination of the urine. This should be done macroscopically, microscopically and chemically. These means afford us valuable aids in diagnosticating the disease.

By reason of the difference in the character of the blood present in the urine we make two divisions of hematuria, namely, *hematuria* pure and simple, and *hemaglobinuria*. In conditions of the first class the blood corpuscles exist intact in the urine; while in conditions of the second class the corpuscles have become disorganized and liberated their hemoglobin, hence the discoloration of the urine.

Occasionally the hemorrhage is so slight that we are unable to detect it macroscopically. In such instances the microscope will generally serve to detect the presence of blood cells; unless their hemoglobin has been liberated (hemoglobinuria), when the

*Pain in the region or high up in the perineum points to hemorrhage from bladder or prostatic urethra.

spectrascope may be necessary to determine their presence.

Apart from the spectrascope and microscope, there are several chemic tests that are both simple and practical. The most popular of these, perhaps, is the one known as Day's test. This test is made by adding a few drops of a fresh alcohol solution of guaiac (It is very well to bear in mind that in patients taking the iodides, the guiacum test, even when blood is absent, gives a greenish blue reaction.—*The Hospital,* Oct. 24, 1908) to the suspected urine and then a small quantity of etheral solution of peroxide of hydrogen. If blood be present a blue reaction results. The ordinary spirits of turpentine (old oxidized turpentine is more trustworthy) and tincture of guaiac when added to urine containing blood also give a decided blue tint.

The gross or microscopical appearance of the urine generally furnishes information. The simplest way of conducting this examination is with the three glass test.* The difference in the color of the urine will be more striking if the patient only expels a drachm or so into the first glass, while into the second glass the bulk should be passed, and into the third the few remaining drops still contained in the bladder. If blood be present in the first glass (fluid blood appearing with the first portion of the stream comes from the deep urethra or prostate. If extensive it may discolor, by flowing backwards, the entire contents of the bladder. The urethroscope may be necessary to settle the question. Goldschmidt's urethroscope through which water, under pressure, is used to dilate the urethra, will in such instances serve our purpose best) and not in the second glass of urine the hemorrhage must be coming from some point anterior to the internal sphincter. If present in the second and not in the first glass, we must look to the kidney, the prostate or the bladder. If next we thoroughly irrigate the bladder and the flow returns clear and then after waiting awhile so as to examine the third glass and find hemorrhage, naturally we suspect either the ureter, kidney or its pelvis.

In a general way, it may be stated that if the blood is of a bright hue the condition giving rise to it is usually benign; also that the nearer the hemorrhage to the vesical neck the brighter the

*The seven glass test of H. H. Young, which even goes a step further than Kollman's five glass test, is a combination of the three glass test and the irrigation test. It requires a little more technique, but it is of undoubted value in obscure and selected cases.

color of the urine; while on the other hand, urine containing blood from morbid or malignant processes is generally of a darker color. When the hemorrhage is urethral it is always washed away with the first gush of urine, provided it does not escape independently of urination. If the urethral hemorrhage has coagulated it is generally discharged as a long, pencil-shaped coagulum and the second glass test will flow comparatively clear; while at the end of micturition the cut-off and accelerator urinae muscles will generally force out from the injured part a varying amount of almost pure blood into the third glass.

Hemorrhage from the prostate is apt to contain clots whose general contour and size are suggestive of the prostatic sinus. Such clots are dark and firm, and pure blood is not observed until urination is nearly completed.

If stone in the pelvis of the kidney is causing the hemorrhage. caudate cells may, in addition to red blood cells, be detected with the microscope. Caudate cells, however, lose their significance when the neck of the bladder is diseased, unless we have collected the urine from each kidney separately through *ureteral* catheters directly from the kidneys. In *acute nephritis* the microscope will show blood casts, epithelium from the renal tubules in the form of casts and, usually, caudate epithelium from the pelvis of the kidney.

Hemorrhage from the ureter is occasionally expelled in clots in the shape of angle worms. This is of importance when differentiation between a vesical and renal tumor is to be made.

In order to complete the examination and verify any presumptive diagnosis we have made, it is, of course, necessary to examine the patient himself, since the final examination may greatly change or modify our opinion as regards the true cause of the hemorrhage.

On the contrary, it not infrequently happens that the information brought forth by the foregoing investigation is so clear and to the point that we can diagnosticate, quite clearly, the underlying cause of the hemorrhage solely by exclusion.

1.—The physical examination should include a thorough examination of the patient's physique, his general make-up; an examination of the heart for lesions; the kidney for the presence of pain and tumors; the rectum for hemorrhoids; the prostate for enlargement; the seminal vesicles for inflammation; and also the base of the bladder itself. In addition to the foregoing the pelvic organs, the bladder, the penis, the testicles and the scrotum should be carefully examined. All this had best be done *without* instruments.

After the examination has proceeded thus far, the best plan is to put the patient to bed for a few days in order that we may better study the case further before examining the urethra and bladder with instruments; for there is danger to these patients in acting *too quickly*. This not only gives us time to give careful thought and study to the case, but affords an opportunity to give him absolute rest; while at the same time we put him on what is practically an alkaline diet: bread and milk. Meanwhile his bowels should be thoroughly evacuated by a mercurial and his kidneys flushed with some good spring water, and an urinary antiseptic—oil of eucalyptus and salol, in 10 minim and 10 grain doses, respectively (capsules), or urotropin in 5 grain to 7 1-2 grain doses is given. Cystogen answers very well, but it is about the same thing as urotropin, only its price is higher. It is a rule to which I have seen no exceptions, that patients receiving this preliminary treatment invariably stand the stone searcher, cystoscopy, urethroscopy, and both ureter and bladder catheterism far better than those not receiving it. In conditions of emergency this preparatory treatment cannot be given and, moreover, in emergencies, cystoscopy and urethroscopy are of little or no value.

2.—The emunctories having responded and the patient having rested the patient is now in prime condition for an urethral examination, at least. If the meatus is small meatotomy should be done, so that the meatus will easily admit a 32 (F.) or 34 (F.) soft, flexible bougie à Boule. It will be necessary to use bougies, corresponding in size to the bougies à Boule, not less than every other day for a week or ten days to prevent the meatus from growing up and actually becoming smaller than it was originally; for at best even when the bougies are used regularly, the meatus will generally contract 2 to 4 millimeters. With the bulbous bougies we can detect any organic or spasmodic strictures that may be present.

By introducing the urethroscope the color, lustre, duplicature and striation of the mucous membrane are observed. Besides erosions and organic stricture, the urethroscope will disclose poly-

(*TO BE CONTINUED*).

